# Bacterial Community Characteristics in the Gastrointestinal Tract of Yak (*Bos grunniens*) Fully Grazed on Pasture of the Qinghai-Tibetan Plateau of China

**DOI:** 10.3390/ani11082243

**Published:** 2021-07-30

**Authors:** Xueping Han, Hongjin Liu, Linyong Hu, Na Zhao, Shixiao Xu, Zhijia Lin, Yongwei Chen

**Affiliations:** 1 Northwest Institute of Plateau Biology, Chinese Academy of Sciences, Xining 810008, China; liuhj@nwipb.cas.cn (H.L.); xiangchou812@163.com (L.H.); zhao88na@163.com (N.Z.); 2 Key Laboratory of Adaptation and Evolution of Plateau Biota, Chinese Academy of Sciences, Xining 810008, China; 3 University of Chinese Academy of Sciences, Beijing 100049, China; 4 Technology Extension Service of Animal Husbandry of Qinghai, Xining 810001, China; imnumberone666@sina.com (Z.L.); liuhongjin0408@163.com (Y.C.)

**Keywords:** bacterial, gastrointestinal tract, yak, characteristics, sex, Qinghai–Tibetan Plateau

## Abstract

**Simple Summary:**

The Qinghai–Tibetan plateau is considered as the third Pole of the world and is characterized by low oxygen, high altitude, extreme cold weather and strong ultraviolet radiation. Yak, as the main domestic animals raised on the plateau, play various roles in local herdsmen’s lives by supplying necessities such as meat, milk and fuel. Yak are adapted to the harsh environment on the plateau; microbiota in gut equip the hosts with special abilities including adaptability, as illustrated by numerous research projects. Accordingly, the microbes in the gastrointestinal tract of yak must be characteristically profiled as a strategy to adapt to the environment. However, little is known about the microbial community in whole tract of yak; almost all of reported researches focused on rumen. Therefore, in the current study the bacterial community in the gastrointestinal tract of yak was explored using 16S rDNA amplicon sequencing technology, and the community profiling characteristic in each section was clearly elucidated.

**Abstract:**

In the current research, samples of yak gastrointestinal tracts (GITs) were used to profile the bacterial compositional characteristics using high through-put sequencing technology of 16S RNA amplicon. A total of 6959 OTUs was obtained from 20,799,614 effective tags, among which 751 OTUs were shared by ten sections. A total of 16 known phyla were obtained in all samples—the most abundant phyla were *Firmicutes* (34.58%), *Bacteroidetes* (33.96%) and *Verrucomicrobia* (11.70%). At the genus level, a total of 66 genera were obtained—*Rikenellaceae_RC9_gut_group* (7.24%), *Akkermansia* (6.32%) and *Ruminococcaceae*_UCG-005 (6.14%) were the most abundant. Species of Observed (Sob), Shannon and Chao values of the Stomach were the greatest, followed by the large intestine, while small intestine had the lowest diversity (*p* < 0.05). *Bacteroidete* were more abundant in sections from rumen to duodenum; while Firmicutes were the most abundant in sections from jejunum. ABC transporters (7.82%), Aminoacyl-tRNA biosynthesis (4.85%) and Purine metabolism (3.77%) were the most abundant level-3 pathways in all samples. The results of associated correlation analysis indicated that rectum samples might be used as an estimator of rumen bacterial communities and fermentation. The results of this research enrich the current knowledge about the unique animals of the QTP and extend our insight into GITs microecology of various animals.

## 1. Introduction

Micro-organisms in animals’ GITs provide digestive abilities via the degradation of complex compounds in various feed materials; especially those invisible organisms in ruminant animals’ rumens that supply the host with the capacity to degrade plant fibers by producing degrading-related enzymes which cannot be secreted by the host [1,2]. The products of degradation are assorted short chain volatile fatty acids (VFAs), which are absorbed by the host to generate the main sources of energy for growth and reproduction [3]. Meanwhile, the microbes fluxed into later parts of the GIT are digested as microbial proteins. The rumen, as the most important organ in the GITs, is the main place of plant fiber degradation, and intensive studies were conducted to investigate its microbial communities, such as microbial characteristics and variations under different environmental factors, e.g., host genetics, diets, feeding regime and age [4,5,6,7,8]. The studies provided insights into the rumen micro-ecology and generated practical results that improved the performance of ruminant animals’ production. Additionally, the rectum was the second most extensively studied focus due to the convenience of sample collection. Microbial profiling of the other sections of the GITs were sparsely considered due to sampling difficulties [9,10]. Each section of GITs plays a different role in the feed conversion as each of them house characteristic microbes which compete for space and nutrients to grow [11]. GITs microbes assisted GITs inner homeostasis, immune response, digestion, physiology and host disease treatment [12]. Microbial diversity along the GIT has been researched in humans, rodents, horses, bison and sheep [11,13,14,15,16]. Yaks, as the majority of the livestock living om the QTP, with an average altitude higher than 3000 m, grazed on grassland all year, which led poor living conditions due to the low quality of grasses and scarcity of food during the cold seasons [17]. As a result, the bacterial composition in the GITs of yaks might have some unique characteristics due to the kinds of specialty of the host and its living environments. While bacterial communities in the GITs of other animals have been subjected to changes due to the rapid development of modern technologies and the abuse of antibiotics, conversely, yaks in native areas are still fed under a traditional regime, which might have lasted for hundreds or thousands of years, and have a bacterial composition that has been kept relatively stable and that could be used as an ideal animal model to study microbial communities and as a mirror of history [18,19]. However, studies of the yak bacterial community have been limited to the study of rumens in different seasons and under different grazing regimes [20,21]. Besides, sex as an important animal trait, and yet few studies considered it as a factor in shaping the bacterial composition of ruminants’ GITs. In the present study, three related scientific issues are hypothesized. Firstly, there are characteristic bacterial compositions for each GITs section and sampled GITs sections might be divided into three regions based on their bacterial profiling. Secondly, the bacterial community differs between sexes. Thirdly, it is possible that rectum samples might be used to be an estimator of the rumen’s fermentation.

## 2. Materials and Methods

### 2.1. Animals and Experimental Design

A total of 5 male yaks (292.0 ± 30.3 kg) and 5 female yaks (268.0 ± 22.8 kg) at age of 45–50 months, grazing on same grassland of the Henan County of Qinghai, China, were commercially slaughtered at a local abattoir on October 2018. No antibiotics were applied within the 3 months before sampling. No animal was specifically slaughtered for this work. All animal care procedures were consistent with the guidelines from the Institution of Animal Care and the Ethics Committee of the Northwest Institute of Plateau Biology, Chinese Academy of Sciences (NWIPB20160302) and this study was approved by the Ethics Committee. After collection, the samples were strictly processed in accordance with the guidelines of NWIPB.

### 2.2. Sampling and Measurements

The digestive organs including stomach, small intestine and large intestine were taken out from the middle body immediately after the animal being slaughtered. Lumen contents samples (2 mL) from ten sections belonging to three functional regions were placed into sterilized freezing tubes using sterilized spoons by making a small cut on the main body of each section, and collected samples were stored in liquid nitrogen for DNA extraction. Sampling organs were rumen (I), reticulum (II), omasum (III), abomasum (IV) of stomach (ST), duodenum (V), jejunum (VI), ileum (VII) of small intestinal (SI) and caecum (VIII), colon (IX) and rectum (X) of large intestinal (LI). In total, 10 samples were collected from each section but only 8 content samples of abomasum and rectum were collected from the male yaks only due to breakage of tubes during freezing. After filtration through 4 layers of sterilized gauze, approximately 65 mL of rumen liquid were taken and then separated into two samples; one sample (15 mL) was immediately used to measure the pH value and the other one (50 mL) used for assessment of rumen fermentation parameters, including ammonia nitrogen (NH_3_-N) and volatile fatty acids (VFAs).

### 2.3. Chemical Analysis

The ruminal liquid pH was measured using a portable pH meter (PHSJ-3F; Precision Instruments Company, Shanghai, China), immediately after sample collection. The VFAs measurements were performed following the method used by Liu et al. (2019). 

### 2.4. DNA Extraction and PCR Amplification

Microbial DNA was extracted using the HiPure Soil DNA Kits (Magen, Guangzhou, China) and according to manufacturer’s protocols. The 16S rRNA V3-V4 regions of the ribosomal RNA gene were amplified by PCR (95 °C for 2 min, followed by 27 cycles at 98 °C for 10 s, 62 °C for 30 s, and 68 °C for 30 s and a final extension at 68 °C for 10 min) using the primers 341F: CCTACGGGNGGCWGCAG and 806R: GGACTACHVGGGTATCTAAT [22], and where the barcode is an eight-base unique sequence for each sample. The PCR reactions were performed in triplicate using a 50 μL mixture containing 5 μL of 10 × KOD Buffer, 5 μL of 2.5 mM dNTPs, 1.5 μL of each primer (5 μM), 1 μL of KOD Polymerase and 100 ng of template DNA. Illumina Hiseq 2500 sequencing Amplicons were extracted from 2% agarose gels and purified using the AxyPrep DNA Gel Extraction Kit (Axygen Biosciences, Union City, CA, USA), according to the manufacturer’s instructions, and quantified using ABI StepOnePlus Real-Time PCR System (Life Technologies, Foster City, CA, USA). The purified amplicons were pooled in equimolar and paired end sequenced (2 × 250) on an Illumina platform according to the standard protocols. The sequencing data of each sample are available at accession No. SRR10613946 to SRR10614043 on Sequence Read Archive (SRA) of National Center of Biotechnology Information (NCBI).

### 2.5. Bioinformatics Analysis

Raw data containing adapters or low-quality reads would affect the following assembly and analysis. Thus, to otain high quality clean reads, raw reads were further filtered according to the following rules using FASTP [23]. Paired end clean reads were merged as raw tags using FLASH (version 0.18.0) (http://ccb.jhu.edu/software/FLASH/, Accessed on 8 May 2021) with a minimum overlap of 10bp and mismatch error rates of 2%. Noisy sequences of raw tags were filtered by QIIME [24] (version 1.9.1) pipeline under specific filtering conditions to obtain high-quality clean tags. The clean tags were searched against the reference database (http://drive5.com/uchime/uchime_download.html, Accessed on 8 May 2021) to perform reference-based chimera checking using UCHIME algorithm (http://www.drive5.com/usearch/manual/uchime_algo.html, Accessed on 8 May 2021). All chimeric tags were removed, and the final effective tags were used for further analysis. The effective tags were clustered into operational taxonomic units (OTUs) of ≥97% similarity using UPARSE pipeline (version 9.2.64) [25]. The tag sequence with the highest abundance was selected as a representative sequence within each cluster. Between groups, Venn analysis was performed in R project (version 1.6.16) to identify unique and common OTUs. The representative sequences were classified into organisms by a naive Bayesian model using the RDP classifier [26] (version 2.2) based on the SILVA database [27] (https://www.arb-silva.de/, Accessed on 8 May 2021). Chao1, Simpson and all other alpha diversity indexes were calculated in QIIME. The OTU rarefaction curve was plotted in QIIME. PCoA (principal coordinates analysis) were calculated and plotted in R project based on the Unweight Unifrac distance. The gene prediction was performed using TAX4FUN [28] and KEGG database.

### 2.6. Statistical Analysis

To understand the interaction of GIT microbiota within the stomach, small intestine, and large intestine, phylogenetic molecular ecological networks (pMENs) (http://ieg4.rccc.ou.edu/mena, Accessed on 15 June 2021) were constructed based on Random Matrix Theory (RMT)-based methods. Top 100 genera were presented and the pMENs were calculated with the same threshold (0.35). The network parameters included centralization betweenness (CB), degree (D), and total links, among which, a low level centralization betweenness (CB) signifies a similar bacteria status and a more community stability of the ruminal bacteria. The keystone species were inferred based on the degree of each node. The variance partitioning analysis (VPA) and the abundance heatmap were calculated and plotted in R using the packages R “vegan” and “pheatmap”, respectively. A canonical correlation analysis (CCA) was conducted based on the Canoco 5 software platform. Significance difference of taxonomic abundance between sections or regions were checked using ANOVA method, and LSD method was applied to conduct multiple comparison, while Kruskal–Wilk test was applied for comparison of alpha diversity between sections. A heatmap analysis was performed using the OmicShare tool, a free online platform for data analysis (http://www.omicshare.com/tools, Accessed on 15 June 2021). The difference in rumen fermentation parameters between the sexes were tested using the *t*-test method of the SPSS 24.0 software

## 3. Results

### 3.1. Chemical Determination Results

The difference of rumen fermentation parameters between sexes were tested using the *t*-test method of the SPSS 24.0 software package (Table 1). No significant difference was observed (*p* > 0.05); however, the pH value of the female samples tended to be greater than the male ones (*p* = 0.052).

### 3.2. Sequencing and Taxonomic Composition of Bacterial Communities

A total of 22,604,597 raw reads were obtained from sequencing, and 20,799,614 effective tags were generated from all samples after quality control—the average effective ratio was 91.97%. All effective tags were aligned and clustered into OTUs (Operation Taxonomic Units, OTUs) based on a cut-off of between-sequences similarity ≥97%, which yielded 2083 OTUs, among which 482 OTUs were shared by samples from 10 sections in the GIT (Figure S1a). The rarefaction curves of all groups tended to saturate to a plateau at the sequencing depth of 80,000 tags (Figure S1b).

**Figure 1:**
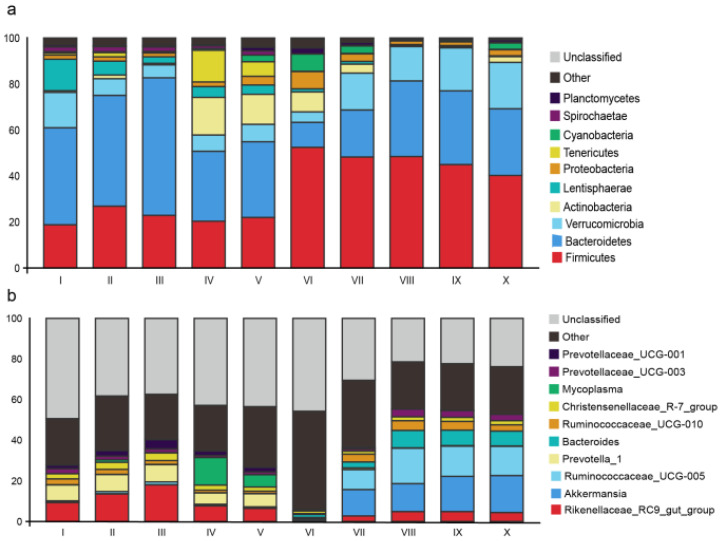
Stack graph shows the bacterial composition based on abundance at the phylum level (**a**) and genus level (**b**) in 10 sections: rumen (I), reticulum (II), omasum (III), abomasum (IV), duodenum (V), jejunum (VI), ileum (VII),caecum (VIII), colon (IX) and rectum (X).

The taxonomic annotation obtained a total of 16 known phyla in all samples (Table S1). At the phylum level, the most abundant bacteria were *Firmicutes* (34.58%), *Bacteroidetes* (33.96%) and *Verrucomicrobia* (11.70%) across all samples (Figure 1a). At the genus level, a total of 66 genera were detected in all samples (Table S2). Among those genera, *Rikenellaceae_RC9_gut_group* (7.24%), *Akkermansia* (6.32%) and *Ruminococcaceae_UCG-005* (6.14%) were the most abundant genera, followed by *Prevotella-1* (3.85%), *Bacteroides* (2.82%), *Ruminococcaceae_UCG-010* (2.63%), *Christensenellaceae_R-7_group* (2.29%), *Mycoplasma* (2.03%), *Prevotellaceae_UCG-003* (1.97%), *Prevotellaceae_UCG-001* (1.56%) and *Paeniclostridium* (1.52%), *Clostridium_sensu_stricto_1* (1.29%), *horsej-a03* (1.26%), *coprostanoligenes_group* (1.25%) and *Ruminococcaceae_NK4A214_group* (1.22%) (Figure 1b).

### 3.3. Diversity and Composition Comparison

To determine bacterial alpha diversity of the gastrointestinal tract, we calculated Species of Observed (Sob), Shannon–Wiener, Chao and Good-coverage indexes. One-way ANOVA package LSD multi-comparison methods of SPSS 24.0 were applied to test the indexes difference among sections of the GITs (Table S3). Except Good-coverage, the other indexes showed the same trend as bacterial communities in the ST region (especially I-III) and were more diverse than the LI region, while the SI region had the lowest diversity (especially VI) in the GITs. The Good-coverage showed no statistical differences among sections (*p* > 0.05). The diversity indexes of each GIT section were compared between the sexes using the SPSS 24.0 software package *t*-test method, The results showed that bacterial communities of the female yaks were more diverse than those of the males, e.g., the sob of I, VI and VIII of the female were significantly greater than the male (*p* < 0.05), and the Chao of the female in IV was greater than the male (*p* < 0.05). There was no significant difference between the sexes in the other sections (*p* > 0.05) (Table S4). 

Chao and Shannon were compared between sections and between the sexes in each region. As shown in Figure S1, the values of Chao and Shannon significantly differed between sections (*p* < 0.05); the diversity of the duodenum (V) was the greatest, followed by the ileum (VII) and the jejunum (VI), which had the lowest diversity in the small intestine. The Shannon value differed between sections in the stomach (*p* < 0.05). The bacterial composition of the abomasum (IV) was the least abundant in the stomach. Both the Chao and Shannon values differed between the sexes in the stomach (*p* < 0.05), but no difference was observed in the other regions (*p* > 0.05).

**Figure 2:**
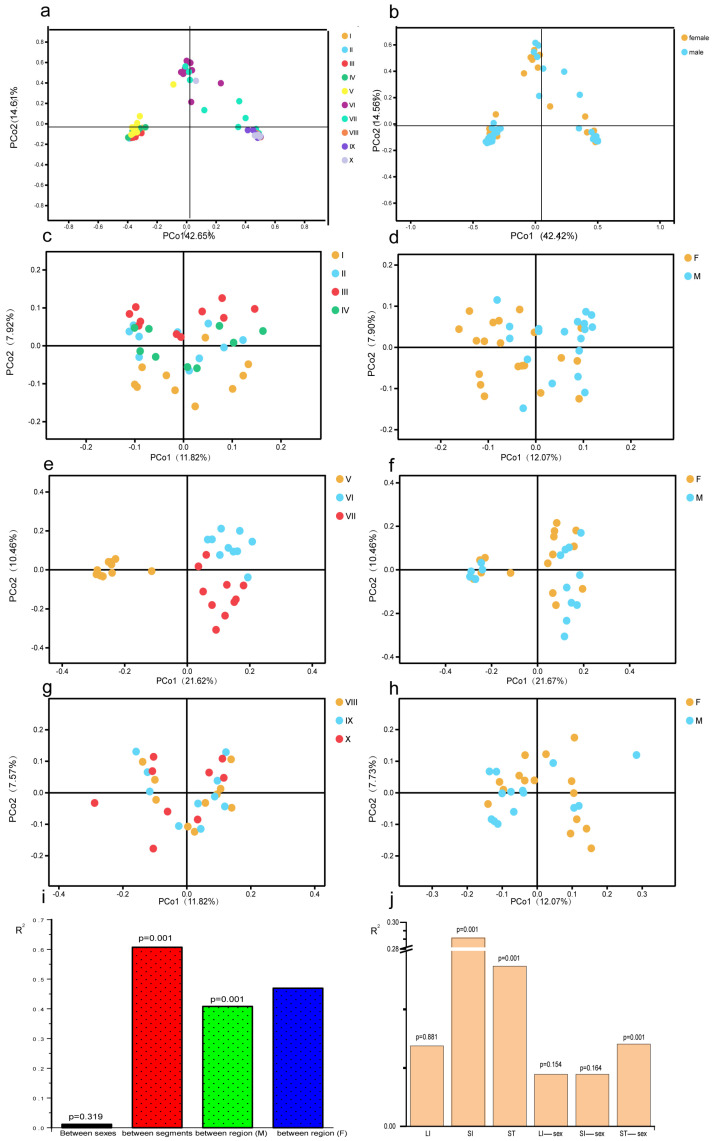
Plot combination illustrates the bacterial composition shift. PCoA plot (**a**,**b**) indicates that all samples are clustered by sections (**a**) and the sexes (**b**) based on Unweighted Unifrac distance. PCoA plot of each region in the GIT (**c**–**h**) based on unweighted Unifrac distance. (**c**–**d**) Samples from 4 sections of stomach; (**e**–**f**) samples from 3 sections of small intestine; (**g**–**h**) samples from 3 sections of large intestine. Bar plots (**i**,**j**) show the Permanova analysis results based on unweighted Unifrac distance, (**i**) plot showing the R2 value of all samples by sections or by sex; (**j**) the R2 value by sections or the sexes in each region, LI is the abbreviation for large intestine, SI is the abbreviation for small intestine and ST is the abbreviation for stomach. The R^2^ value is used to assess the extent of discrepancy between sections or the sexes. The *p*-value above each bar means difference significance, *p* < 0.05 means significantly different.

As shown in Figure 2, the bacterial composition significantly varied between the gut sections, regions or sex, as indicated by PCoA and PERMANOVA (*p* < 0.05). All samples were clustered into three groups as a result of the PCoA analysis. However, samples from V and VII were, respectively, mixed into other clusters, which mightly due to the anatomical connection with VI and VIII. The results were similar regardless of the sex (Figure 2a). Bacterial composition did not differ between the sexes (*p* > 0.05) (Figure 2b). The results of the Permanova analysis suggest that the section was the most important factor shaping the bacterial composition of the GIT, followed by the region, while sex difference was a lesser determinant (Figure 2c). The bacteria composition differences between sections and sexes in each region are shown in PCoA plot and Permanova analysis (Figure 2a–g). The samples separated into clusters according to regions, which were supported by the R^2^ value of Permanova analysis (*p* = 0.001, *p* = 0.001 for stomach and small intestine, respectively); however, no difference was observed between sections in the large intestine (*p* = 0.881). The composition difference between the sexes was significant in the stomach (*p* = 0.001), but no difference was observed in small intestine and large intestine (*p* > 0.05).

**Figure 3:**
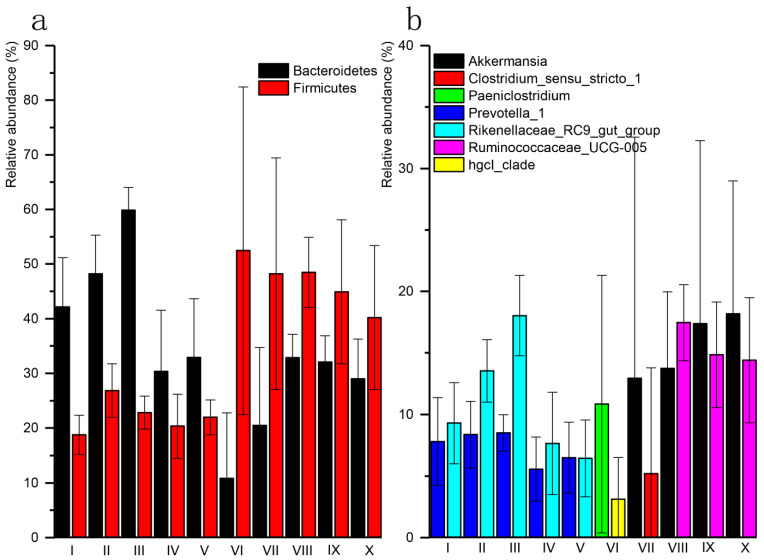
Bar plot shows the most abundant two phylum (**a**) and genus (**b**) based on relative abundance in 10 sections: rumen (I), reticulum (II), omasum (III), abomasum (IV), duodenum (V), jejunum (VI), ileum (VII), caecum (VIII), colon (IX) and rectum (X) in the GIT of yaks.

The phylum *Bacteroidete* was more abundant in sections I to V; while, *Firmicutes* was more abundant in sections VI to X (Figure 3a). The abundance of other phyla varied among sections (Table S5). At the genus level, *Prevotella_1* and *Rikenellaceae_RC9_gut_groups* of *Bacteroidetes* were the most abundant in sections I to V. In VI, the most abundant genera were *Paeniclostridium* (*Firmicutes*) and *hgcI_clade* (*Actinobacteria*), while *Akkermansia* (*Verrucomicrobia*) and *Clostridium_sensu_stricto_1* (*Firmicutes*) were the most abundant in VII. For the sections VIII to X, the most abundant genera were *Ruminococcaceae_UCG-005* and *Akkermansia* of *Firmicutes* (Figure 3b).

### 3.4. Predicted Functions in GIT Regions between the Sexes

**Figure 4:**
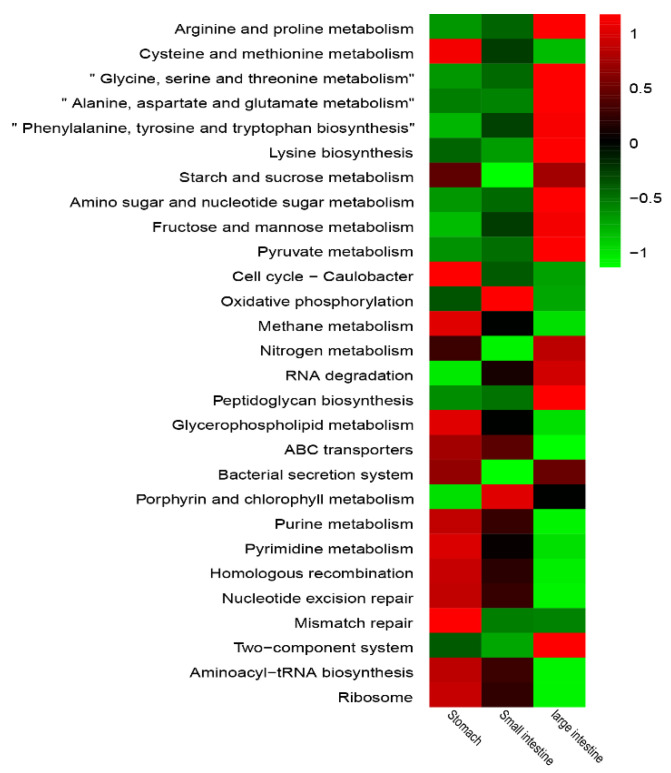
Heatmap shows enrichment of the predicted function genes (relative abundance > 1%) at level-3 between three regions (stomach, small intestine and large intestine) in the GIT of yaks. The relative abundance of each gene is shown in the heatmap and normalized by row.

The Tax4FUN method was applied to predict KEGG function genes of the bacterial communities in the GIT of yaks. ABC transporters (7.82%), Aminoacyl-tRNA biosynthesis (4.85%) and Purine metabolism (3.77%) were the most abundant level-3 function genes in all GITs regions of yaks (Table S5). As shown in Figure 4 and Table S5, the number of functional genes enriched in the stomach was higher than in other regions, followed by the large intestine and the small intestine, which had the least. Each region of the GITs had its own main functions, as shown by the heatmap, where functional genes with higher abundances presented lower abundances in other regions. Cellular processes (cell cycle-Caulobacter), environmental information processing (bacterial secretion system), genetic information processing (mismatch repair), metabolism (cysteine and methionine metabolism, methane metabolism, glycerophospholipid metabolism, purine metabolism and pyrimidine metabolism), were significantly abundant in the stomach compared to other two regions (*p* < 0.05). Metabolism (porphyrin and chlorophyll metabolism) was obviously abundant in small intestine (*p* < 0.05). Glycine, serine, threonine, alanine, aspartate, glutamate, phenylalanine and tyrosine metabolisms, tryptophan biosynthesis, amino sugar and nucleotide sugar metabolism, fructose and mannose metabolism, pyruvate metabolism and peptidoglycan biosynthesis, were mostly abundant in the large intestine (*p* < 0.05) and were mainly related to carbohydrates.

### 3.5. Correlation Analysis between Taxonomic Units and Fermentation Parameters

**Figure 5:**
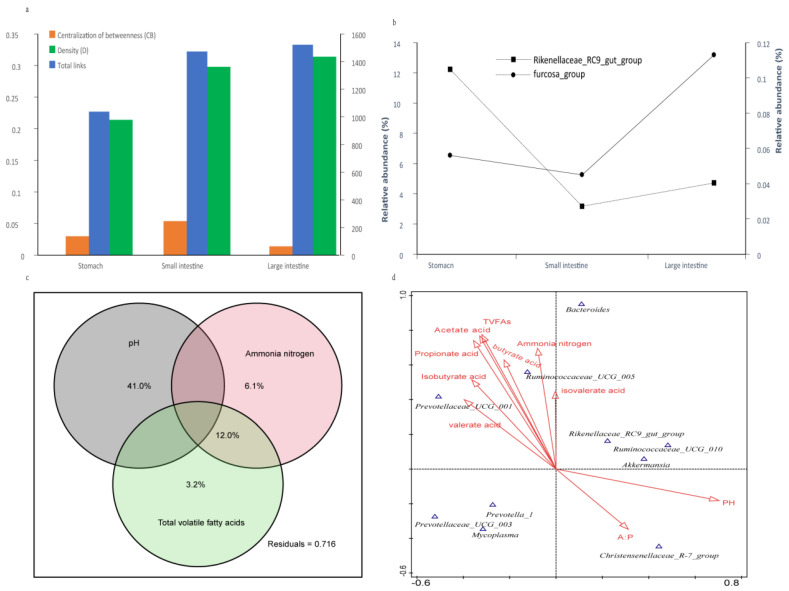
Correlation analysis between genus profiling and fermentation parameters. (**a**) Network parameters in three regions of GIT. All parameters were calculated at the same cutoff of 0.32; (**b**) Variation of the relative abundance of the putative keystone genera in three regions of the GIT; (**c**) VPA showing the most important fermentation parameters that impact the rumen genus profiling; (**d**) CCA of rumen genus profiling and fermentation parameters.

The total links and density (D) of the large intestine were greater than those of the small intestine, while the stomach had the smallest amount. The centralization of betweenness (CB) of the small intestine was the greatest, while the large intestine had the smallest (Figure 5a). The putative keystone genera were calculated based on the number of network degrees; the *furcosa_*group was the keystone genus in the stomach and large intestine, and its relative abundance peaked to 0.11% in the large intestine and dropped to 0.06% and 0.05% in the stomach and small intestine, respectively. *Rikenellaceae_RC9_*gut_group was the keystone genus in the small intestine, and its relative abundance was 12.23% in the stomach, which sharply fell to 3.17% and 4.72% in the small and large intestines, respectively (Figure 5b). The pH value added the biggest influence on the rumen bacterial composition, as shown by the 41.0% compositional variation (Figure 5c). The pH value negatively correlated with the fermentation parameters and positively correlated with the ratio of A:P. Additionally, the pH value significantly correlated with the relative abundance of the *Akkermansia*, *Ruminococcaceae_UCG-010*, *Rikenellaceae_RC9_* and *Christensenellaceae_R-7_*gut groups (Figure 5d). 

## 4. Discussion

Traditional molecular methods were extensively used to describe the microbial community in sections of animals’ GITs, such as wild gorilla, pigs, chicken, mice and dairy cows [29,30,31,32,33,34]. These methods were rapidly replaced by high throughput sequencing technology due to their advantages of higher efficiency and absolution advantages. The new method was used to deeply elucidate GITs micro-ecology in humans and animals, such as rodents, horses, bison and sheep [11,13,14,15,16]. Yak is a unique domesticated animal which is adapted to harsh, cold and low oxygen environment. Several studies paid attention to rumen microbiota under different environmental factors, such as seasons, raising regime [3,20] and rapid fattening regimes [35]. However, no studies were conducted to investigate the bacterial communities in the GITs of yaks that grazed on grassland over the year, and the bacterial composition shift between sexes. The results of PCoA analysis showed that the bacterial composition of stomach was similar. The last three sections of the large intestine had parallel bacterial profile with the jejunum composed of the third cluster; while the bacterial composition of other two sections of the small intestine were separated from the jejunum. Previous studies on the microbial communities in the GITs showed similar trends [15,16]. When the samples from adjacent parts of the GIT were clustered together, the microbial community composition significantly differed between regions, but the composition of samples in the same region was similar [11,16,36]. As commonly known, the GITs was divided into three functional regions, namely stomach, small intestine and large intestine. Interestingly, the bacterial composition supported the classic physiology results, which were also supported by the results of PERMANOVA analysis. Meanwhile, the bacterial composition in the rumen, reticulum and caecum differed between sexes. Previous studies have shown that diet had more effect on microbial composition than age, sex and location [37,38]. The gut microbiota of the moose varied with age, weight and location, but not with sex [39]. Li et al. reported that the rumen bacterial composition varied with the forage growth stage, but no difference was observed between sexes [3]. However, some studies provided evidence of the existence of a microbial difference between the sexes [40,41]. Reports about the GITs microbial composition were inconsistent, possibly due to the noise introduced by environmental variates, such as age, diet and host’s genetics [42,43,44]. The results of a research project conducted under a specific genetic background showed a difference between the sexes, which became prominent [41]. Sex exerted less impact on the bacterial composition compared to other factors, such as the host and diet. These results were confirmed by a previous research that showed that sex was the 10th most dominant factor among the 69 factors associated with microbial communities [45]. Additionally, another research paper reported that sex explained 11.6% of the variance of the bacterial composition [46]. The results of the present research indicated that section of GITs was a considerably more important a factor in shaping GIT bacterial composition than the sex of the host animals. The above results show that the second hypothesis was true.

In the current research, we found that the stomach bacterial community was the most abundant, followed by the large intestine, as also shown by Sobs, Shannon and Chao; while, the small intestine presented the lowest diversity. Previous studies on GIT microbes demonstrated similar results [11,16,43]. The small intestine had the lowest abundance due to two key reasons. Firstly, the peristaltic movement gave a short retention time in the section and less time for the bacteria to proliferate [47,48]. Secondly, the small intestine had a relatively lower pH value due to the impact of abomasum, whose pH value ranged from 2 to 4; meanwhile, the bile acid from the gall bladder and the pancreatic enzymes were received by the region [49] and presented a considerably harsher environment for the majority of microbes. The stomach and caecum bacterial diversity in female yaks were more diverse than in males. More recent studies indicated that women have a more diverse gut microbiome than men, and sex shapes the composition of the gut microbiome after puberty [50,51,52]. Takagi et al. reported that there was no significant alpha diversity difference between the sexes in the healthy Japanese gut [53]. The alpha diversity difference between the sexes showed that the stomach bacteria of female yaks were more diverse than in the males, but no discrepancy was observed in other regions.

The present study found that phylum *Firmicutes*, *Bacteroidetes* and *Verrucomicrobia* predominated in all samples in the GITs of yak. Studies on the microbial composition in ruminants GITs found that *Firmicutes, Bacteroidetes* and *Proteobacteria* were predominant in all samples of goats and calves [54,55]. A study on yak rumen bacterial composition confirmed our results [21]. The high abundance of Verrucomicrobia was probably due to the genetic discrepancy between the yak and other ruminal animals. However, previous research [35] reported that *Bacteroidetes*, *Firmicutes* and *Proteobacteria* were predominant in the rumen of yaks and this variation might be due to the difference of amplicon region in that report with our current work. Researchers reported that *Verrucomicrobia* contained a broad repertoire of glycoside hydrolyze enzymes and played a key role in the degradation of polysaccharide and cellobiose [16,56,57,58]. When it came to sections, *Bacteroidetes* were the most abundant in the four sections of the stomach and duodenum; while *Firmicutes* predominated for the jejunum to rectum section, Zeng et al. reported that the abundance of *Bacteroidetes* was higher in the stomach and large intestine compared to the small intestine [16]. Similar results reported that *Bacteroidetes* in rumen, reticulum and omasum of the bovines were greater [59]. The difference between our results and previous works may be introduced by the different host genetics or environmental factors that were applied to the hosts. *Bacteroidetes* functioned in the digestion of complex carbohydrates [60], while *Firmicutes* was composed of fibrolytic and cellulolytic bacterial genera [61]. In this research, *Prevotella_1* was the most abundant in the sections of the stomach and duodenum. In the jejunum, the most abundant genus was *Paeniclostridium*, while *Akkermansia* was the most abundant in the ileum. In the large intestine, the most abundant genera were *Ruminococcaceae_UCG-005* and *Akkermansia*. *Prevotella* played a role in proteins’ utilization in the rumen and were reported to work in conjunction with cellulolytic bacteria to degrade hemicellulose [62]. *Ruminococcus* aided in the digestion and metabolism of dietary fiber [6]. The above results proved that the first hypothesis was true and that each part of the GIT has its own bacterial composition characteristics.

ABC transporters (7.82%), aminoacyl-tRNA biosynthesis (4.85%) and purine metabolism (3.77%) were the most abundant metabolism pathway in the GITs of yaks. It was reported that ABC transporters were enriched in the rumen of yaks and sheep on the QTP [63]. ABC transporters protect the host from being hurt due to the intake of poisonous substrates [64], which might be an explanation of yaks’ adaptability to harsh environment on the QTP. Most level-3 pathways of cellular processes, environmental information processing and genetic information processing, were abundant in the stomach and small intestine, especially in the latter; while, the large intestine presented its main functions in amino acid metabolism and carbohydrate metabolism. Besides, the stomach was the main location of nucleotide metabolism, lipid metabolism, energy metabolism, starch and sucrose metabolisms, and arginine and proline metabolisms.

It was reported that the rumen retained a healthy and stable inner ecological environment with pH ranging from 6.2 to 7.0 [65]. The pH values were 6.52 and 6.44 for female and male yaks in the current research, respectively, which meant that the rumen fermentation of fully grazed yak was normal and stable, with no difference between the sexes. Many factors affected the rumen pH, such as the ratio of dietary flour and starches, the rumen sample collection time interval with concentrated feed intake, and also other factors [66,67]. The rumen fermentation was mainly affected by the feed ingredients; the high content of protein and soluble carbohydrates in the feed that induced high VFAs concentrations [68]. It was reported that the concentrations of VFAs in the rumen of domestic animals in QTP are greater than those in ruminants that are raised in lower altitude areas [69], which may be an adaptative strategy due to the special environment on the plateau. The interaction between genera in the large intestine and the small intestine was more complicated than that in the stomach, which might contradict our common cognitions that the key fermentation roles of the feed were played by ruminal microbes. However, this research just focused on bacterial interaction without considering other microbes, such as fungi, and protozoa; thus, the results need to be further supplemented in the future. The *furcosa_group* that is member of the phylum *Firmicutes*, was the inferred keystone genus in the stomach and the large intestine. The *furcosa_group* did not predominate in the GIT, a result that is consistent with previous studies [70] that showed that the keystone genus was not always the predominant genus. *Rikenellaceae_RC9_gut_group* was the inferred keystone genus in the small intestine and the *Rikenellaceae_RC9_gut_group* was the main member of the phylum *Bacteroidetes*. It was reported that the presence of *Rikenellaceae* in the gut correlates with resistance to the development of colitis, through its ability to limit inflammation by stimulating T-regulatory cells’ differentiation [71]. Variance partitioning analysis (VPA) showed that the pH value of the rumen contents exerts the greatest impact on the ruminal bacterial composition, and that the pH value negatively correlates with the rumen fermentation parameters, while it is positively associated with the ratio of A:P and according to the results of CCA. All contents of VFA were positively associated with each other. VFAs are the byproducts of rumen fermentation and take up most of the energy sources of ruminants. 

## 5. Conclusions

As the first research on the bacterial composition of the whole GITs of yaks grazed on the QTP, we obtained some intriguing results. As hypothesized, we first showed that each section had its characteristic taxonomic profiling; the phylum *Bacteroidetes* was more abundant in sections from the rumen to the duodenum, while *Firmicutes* was more abundant in sections from the jejunum to the rectum. At the genus level, *Prevotella_1* was the most abundant in the sections from the rumen to duodenum, in jejunum, the most abundant genera were *Paeniclostridium*, while *Akkermansia* was the most abundant in the ileum. For the sections from the caecum to the rectum, the most abundant genera were Ruminococcaceae_UCG-005. Secondly, bacterial difference between sexes was found in several sections, but the different sections had a greater impact on shaping the bacterial composition in the GITs of yaks than their sexes. pH value had the greatest impact on the rumen bacterial composition.

## Figures and Tables

**Table 1:** Rumen fermentation parameters of male and female yaks.

Items	Female Yaks	Male Yaks	*p*-Value
pH	6.52 ± 0.06	6.44 ± 0.04	0.052
Ammonia nitrogen(mg/L)	104.60 ± 46.15	109.50 ± 20.92	0.834
Acetate (mmol/L)	14.00 ± 5.87	21.43 ± 12.21	0.268
Propionate (mmol/L)	2.87 ± 1.34	4.44 ± 2.54	0.268
Isobutyrate (mmol/L)	1.02 ± 0.46	1.30 ± 0.55	0.405
butyrate (mmol/L)	2.42 ± 2.02	2.87 ± 1.69	0.710
isovalerate (mmol/L)	1.30 ± 1.56	0.98 ± 0.61	0.682
valerate (mmol/L)	0.79 ± 0.34	1.02 ± 0.38	0.354
Total volatile fatty acids (mmol/L)	21.79 ± 9.59	32.03 ± 17.60	0.296
Acetate to propionate ratio (A:P)	5.05 ± 0.97	4.9 ± 0.75	0.789

## Data Availability

The sequencing data of each sample are available by the accession No. SRR10613946 to SRR10614043 on Sequence Read Archive (SRA) of National Center of Biotechnology Information (NCBI).

## Supplementary Materials

The following are available online at https://www.mdpi.com/article/10.3390/ani11082243/s1, Figure S1: bar-plot of Chao and Shannon index comparison between sections of each region and between the sexes within each region; Table S1: Phyla abundance in each sections in the gastrointestinal tract of yaks (%); Table S2: Predominated four genera in each section of GIT of yaks; Table S3: Difference of alpha diversity value among sections in the gastrointestinal tract of yaks; Table S4: Difference of alpha diversity among sections in the gastrointestinal tract of yaks and between the sexes within each section; Table S5: Predicted functional genes in each region of GIT of yaks with a relative abundance > 1%.
