# Supplementary material for: Bacterial Community Characteristics in the Gastrointestinal Tract of Yak (Bos grunniens) Fully Grazed on Pasture of the Qinghai-Tibetan Plateau of China
Source: Animals (Basel). 2021 Jul 30;11(8):2243. doi: 10.3390/ani11082243 (PMC8388508; doi:10.3390/ani11082243)
Supplement: Supplementary file 1 [file animals-11-02243-s001.zip › animals-1307468-supplementary/Supplementary/Legend of Fig.S1.pdf]

Fig. S1 bar-plot of Chao and Shannon index comparison between segments of each region and between the sexes within each region. a-c) indicated difference of Chao value between segments in each region, namely, large intestine (a), small intestine (b) and stomach (c); d-f) indicated difference of Chao value between the sex in each region, namely, large intestine (d), small intestine (e) and stomach (f). g-i) indicated difference of Shannon value between segments in each segment, namely, large intestine (g), small intestine (h) and stomach (i); j-l) indicated difference of Shannon value between the sexes in each region, namely, large intestine (j), small intestine (k) and stomach (l). In each segment, large intestinal included including caecum (I), colon (II) and rectum (III); small intestine included duodenum (I), jejunum (II), ileum (III); stomach included rumen (Ⅰ), reticulum (Ⅱ), omasum (Ⅲ), abomasum (Ⅳ). P-value on the bar indicated difference significance, $p<0.05$ meant significantly different. Difference was tested between segments in each region using Kru-wall method and between the sexes using T-test method.
