# Supplementary figures and images for: Bacterial Community Characteristics in the Gastrointestinal Tract of Yak (Bos grunniens) Fully Grazed on Pasture of the Qinghai-Tibetan Plateau of China
Source: Animals (Basel). 2021 Jul 30;11(8):2243. doi: 10.3390/ani11082243 (PMC8388508; doi:10.3390/ani11082243)

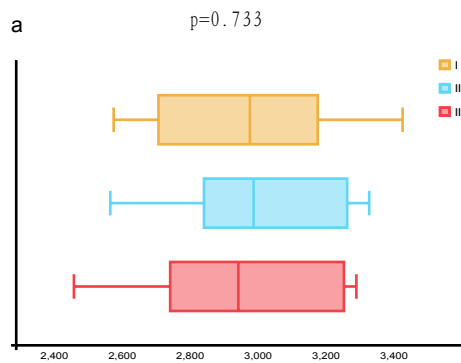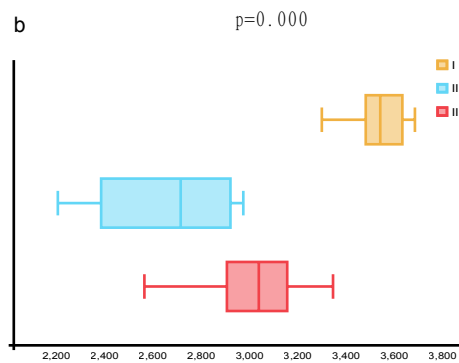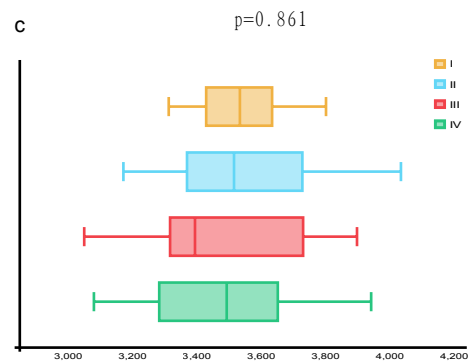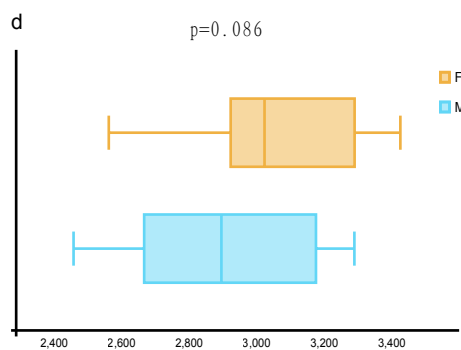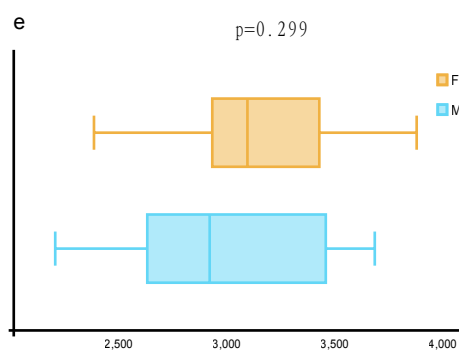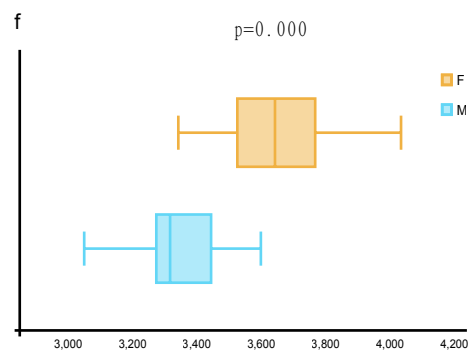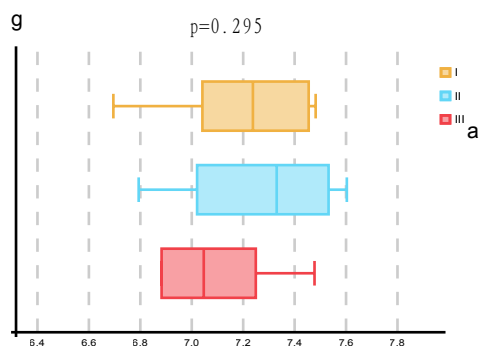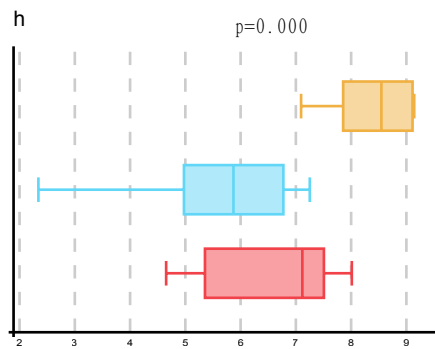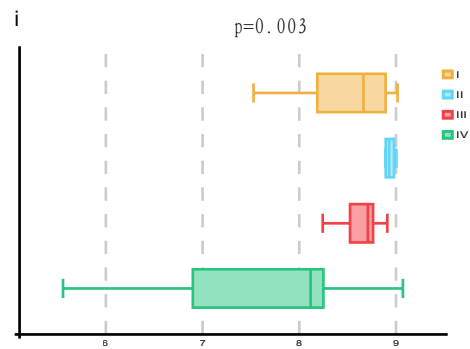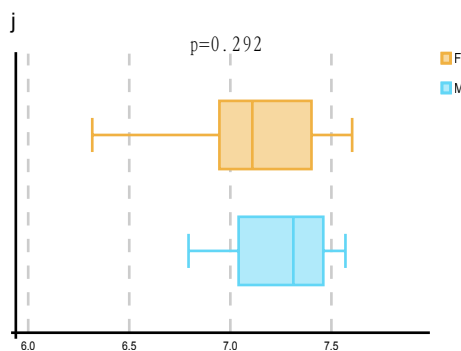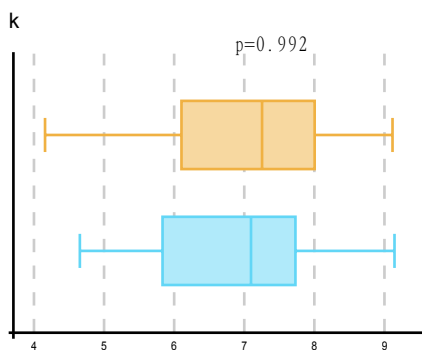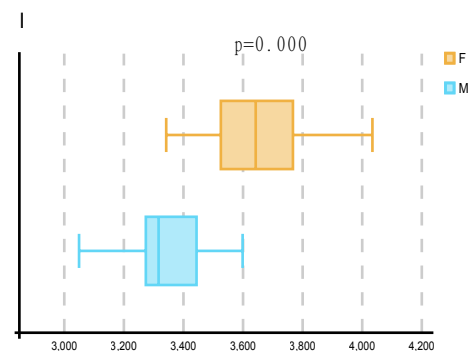

Supplement: Supplementary file 1 [file animals-11-02243-s001.zip › animals-1307468-supplementary/Supplementary/Fig.S1.pdf]
